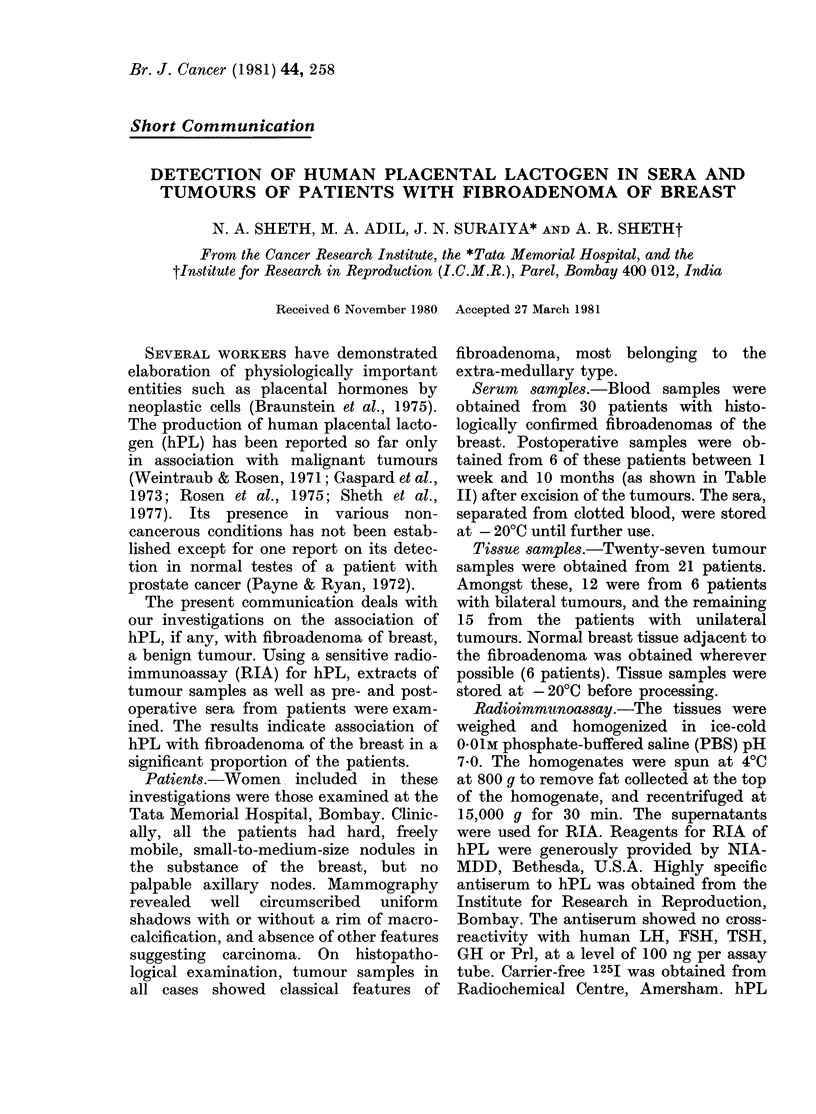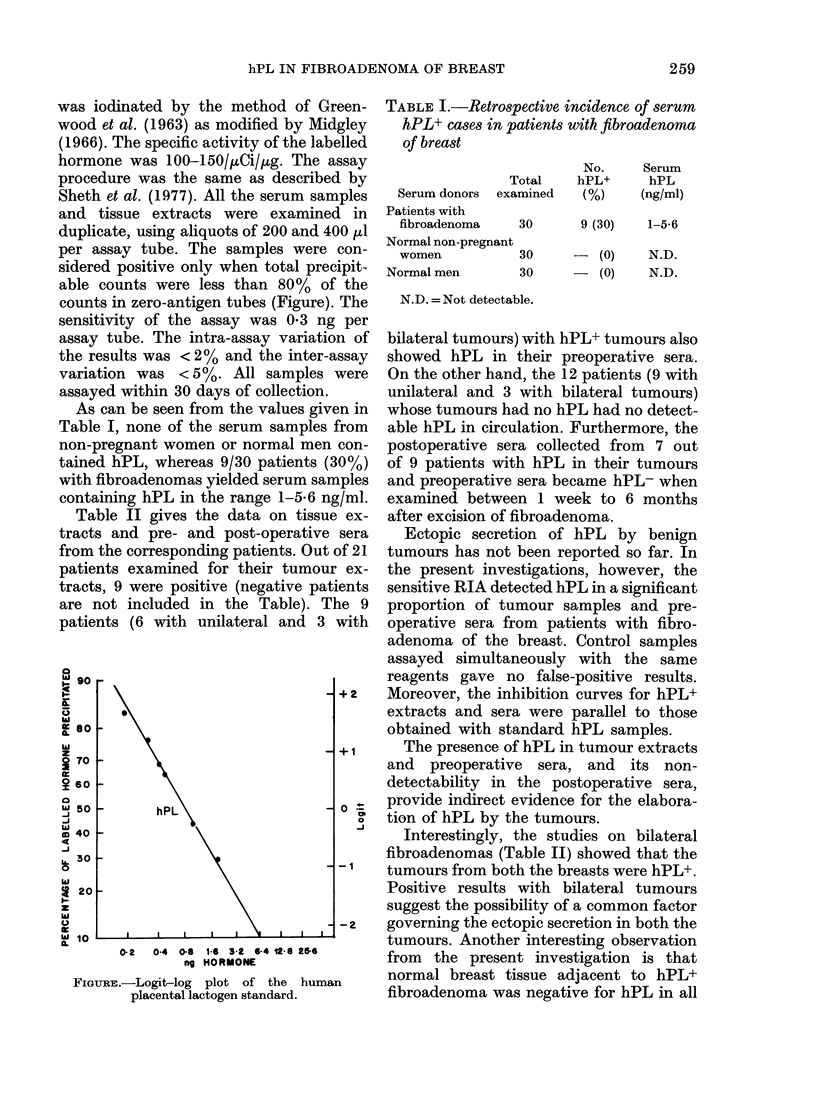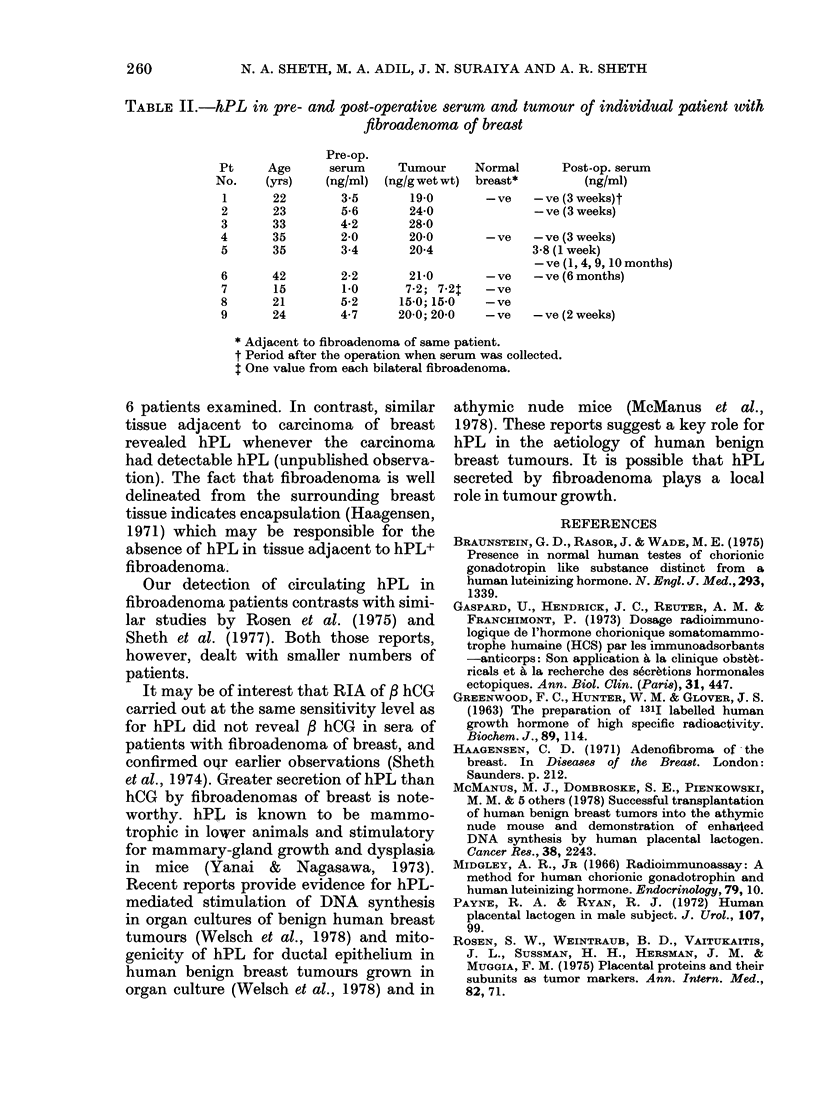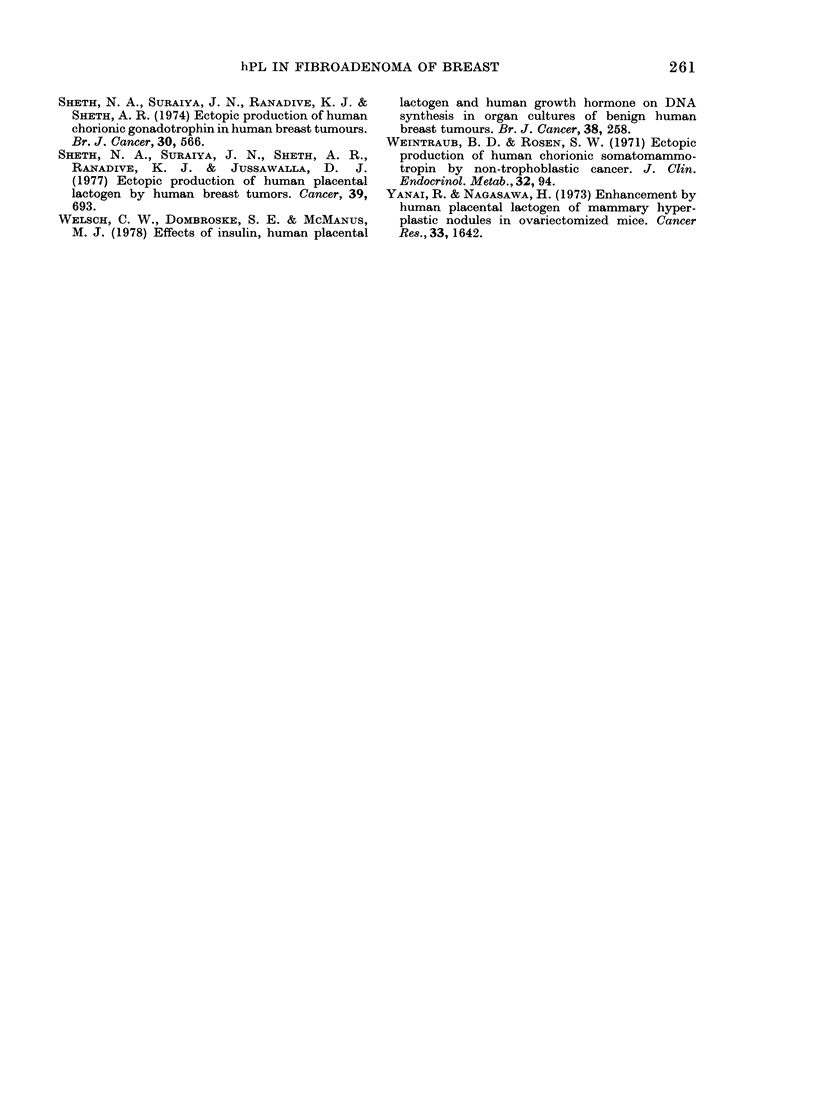# Detection of human placental lactogen in sera and tumours of patients with fibroadenoma of breast.

**DOI:** 10.1038/bjc.1981.177

**Published:** 1981-08

**Authors:** N. A. Sheth, M. A. Adil, J. N. Suraiya, A. R. Sheth


*Br. J. Cancer* (1981) **44**, 258

*Short Communication*

### DETECTION OF HUMAN PLACENTAL LACTOGEN IN SERA AND TUMOURS OF PATIENTS WITH FIBROADENOMA OF BREAST

N. A. SHETH, M. A. ADIL, J. N. SURAIYA* AND A. R. SHETH†

*From the Cancer Research Institute, the *Tata Memorial Hospital, and the †Institute for Research in Reproduction (I.C.M.R.), Parel, Bombay 400 012, India*

Received 6 November 1980 Accepted 27 March 1981

SEVERAL WORKERS have demonstrated elaboration of physiologically important entities such as placental hormones by neoplastic cells (Braunstein *et al.*, 1975). The production of human placental lactogen (hPL) has been reported so far only in association with malignant tumours (Weintraub & Rosen, 1971; Gaspard *et al.*, 1973; Rosen *et al.*, 1975; Sheth *et al.*, 1977). Its presence in various non-cancerous conditions has not been established except for one report on its detection in normal testes of a patient with prostate cancer (Payne & Ryan, 1972).

The present communication deals with our investigations on the association of hPL, if any, with fibroadenoma of breast, a benign tumour. Using a sensitive radioimmunoassay (RIA) for hPL, extracts of tumour samples as well as pre- and post-operative sera from patients were examined. The results indicate association of hPL with fibroadenoma of the breast in a significant proportion of the patients.

*Patients.*—Women included in these investigations were those examined at the Tata Memorial Hospital, Bombay. Clinically, all the patients had hard, freely mobile, small-to-medium-size nodules in the substance of the breast, but no palpable axillary nodes. Mammography revealed well circumscribed uniform shadows with or without a rim of macrocalcification, and absence of other features suggesting carcinoma. On histopathological examination, tumour samples in all cases showed classical features of fibroadenoma, most belonging to the extra-medullary type.

*Serum samples.*—Blood samples were obtained from 30 patients with histologically confirmed fibroadenomas of the breast. Postoperative samples were obtained from 6 of these patients between 1 week and 10 months (as shown in Table II) after excision of the tumours. The sera, separated from clotted blood, were stored at −20°C until further use.

*Tissue samples.*—Twenty-seven tumour samples were obtained from 21 patients. Amongst these, 12 were from 6 patients with bilateral tumours, and the remaining 15 from the patients with unilateral tumours. Normal breast tissue adjacent to the fibroadenoma was obtained wherever possible (6 patients). Tissue samples were stored at −20°C before processing.

*Radioimmunoassay.*—The tissues were weighed and homogenized in ice-cold 0·01M phosphate-buffered saline (PBS) pH 7·0. The homogenates were spun at 4°C at 800 *g* to remove fat collected at the top of the homogenate, and recentrifuged at 15,000 *g* for 30 min. The supernatants were used for RIA. Reagents for RIA of hPL were generously provided by NIAMDD, Bethesda, U.S.A. Highly specific antiserum to hPL was obtained from the Institute for Research in Reproduction, Bombay. The antiserum showed no cross-reactivity with human LH, FSH, TSH, GH or Prl, at a level of 100 ng per assay tube. Carrier-free $^{125}$I was obtained from Radiochemical Centre, Amersham. hPL

was iodinated by the method of Greenwood *et al.* (1963) as modified by Midgley (1966). The specific activity of the labelled hormone was 100–150/$\mu$Ci/$\mu$g. The assay procedure was the same as described by Sheth *et al.* (1977). All the serum samples and tissue extracts were examined in duplicate, using aliquots of 200 and 400 $\mu$l per assay tube. The samples were considered positive only when total precipitable counts were less than 80% of the counts in zero-antigen tubes (Figure). The sensitivity of the assay was 0·3 ng per assay tube. The intra-assay variation of the results was $<2\%$ and the inter-assay variation was $<5\%$. All samples were assayed within 30 days of collection.

As can be seen from the values given in Table I, none of the serum samples from non-pregnant women or normal men contained hPL, whereas 9/30 patients (30%) with fibroadenomas yielded serum samples containing hPL in the range 1–5·6 ng/ml.

Table II gives the data on tissue extracts and pre- and post-operative sera from the corresponding patients. Out of 21 patients examined for their tumour extracts, 9 were positive (negative patients are not included in the Table). The 9 patients (6 with unilateral and 3 with

FIGURE.—Logit–log plot of the human placental lactogen standard.

TABLE I.—*Retrospective incidence of serum hPL⁺ cases in patients with fibroadenoma of breast*

| Serum donors | Total examined | No. $hPL^+$ (%) | Serum hPL (ng/ml) |
|---|---|---|---|
| Patients with fibroadenoma | 30 | 9 (30) | 1–5·6 |
| Normal non-pregnant women | 30 | — (0) | N.D. |
| Normal men | 30 | — (0) | N.D. |

N.D. = Not detectable.

bilateral tumours) with $hPL^+$ tumours also showed hPL in their preoperative sera. On the other hand, the 12 patients (9 with unilateral and 3 with bilateral tumours) whose tumours had no hPL had no detectable hPL in circulation. Furthermore, the postoperative sera collected from 7 out of 9 patients with hPL in their tumours and preoperative sera became $hPL^-$ when examined between 1 week to 6 months after excision of fibroadenoma.

Ectopic secretion of hPL by benign tumours has not been reported so far. In the present investigations, however, the sensitive RIA detected hPL in a significant proportion of tumour samples and preoperative sera from patients with fibroadenoma of the breast. Control samples assayed simultaneously with the same reagents gave no false-positive results. Moreover, the inhibition curves for $hPL^+$ extracts and sera were parallel to those obtained with standard hPL samples.

The presence of hPL in tumour extracts and preoperative sera, and its nondetectability in the postoperative sera, provide indirect evidence for the elaboration of hPL by the tumours.

Interestingly, the studies on bilateral fibroadenomas (Table II) showed that the tumours from both the breasts were $hPL^+$. Positive results with bilateral tumours suggest the possibility of a common factor governing the ectopic secretion in both the tumours. Another interesting observation from the present investigation is that normal breast tissue adjacent to $hPL^+$ fibroadenoma was negative for hPL in all

TABLE II.—*hPL in pre- and post-operative serum and tumour of individual patient with fibroadenoma of breast*

| Pt No. | Age (yrs) | Pre-op. serum (ng/ml) | Tumour (ng/g wet wt) | Normal breast* | Post-op. serum (ng/ml) |
|---|---|---|---|---|---|
| 1 | 22 | 3·5 | 19·0 | –ve | –ve (3 weeks)† |
| 2 | 23 | 5·6 | 24·0 | | –ve (3 weeks) |
| 3 | 33 | 4·2 | 28·0 | | |
| 4 | 35 | 2·0 | 20·0 | –ve | –ve (3 weeks) |
| 5 | 35 | 3·4 | 20·4 | | 3·8 (1 week) –ve (1, 4, 9, 10 months) |
| 6 | 42 | 2·2 | 21·0 | –ve | –ve (6 months) |
| 7 | 15 | 1·0 | 7·2; 7·2‡ | –ve | |
| 8 | 21 | 5·2 | 15·0; 15·0 | –ve | |
| 9 | 24 | 4·7 | 20·0; 20·0 | –ve | –ve (2 weeks) |

* Adjacent to fibroadenoma of same patient.
† Period after the operation when serum was collected.
‡ One value from each bilateral fibroadenoma.

6 patients examined. In contrast, similar tissue adjacent to carcinoma of breast revealed hPL whenever the carcinoma had detectable hPL (unpublished observation). The fact that fibroadenoma is well delineated from the surrounding breast tissue indicates encapsulation (Haagensen, 1971) which may be responsible for the absence of hPL in tissue adjacent to hPL+ fibroadenoma.

Our detection of circulating hPL in fibroadenoma patients contrasts with similar studies by Rosen *et al.* (1975) and Sheth *et al.* (1977). Both those reports, however, dealt with smaller numbers of patients.

It may be of interest that RIA of $\beta$ hCG carried out at the same sensitivity level as for hPL did not reveal $\beta$ hCG in sera of patients with fibroadenoma of breast, and confirmed our earlier observations (Sheth *et al.*, 1974). Greater secretion of hPL than hCG by fibroadenomas of breast is noteworthy. hPL is known to be mammotrophic in lower animals and stimulatory for mammary-gland growth and dysplasia in mice (Yanai & Nagasawa, 1973). Recent reports provide evidence for hPL-mediated stimulation of DNA synthesis in organ cultures of benign human breast tumours (Welsch *et al.*, 1978) and mitogenicity of hPL for ductal epithelium in human benign breast tumours grown in organ culture (Welsch *et al.*, 1978) and in athymic nude mice (McManus *et al.*, 1978). These reports suggest a key role for hPL in the aetiology of human benign breast tumours. It is possible that hPL secreted by fibroadenoma plays a local role in tumour growth.

### REFERENCES

BRAUNSTEIN, G. D., RASOR, J. & WADE, M. E. (1975) Presence in normal human testes of chorionic gonadotropin like substance distinct from a human luteinizing hormone. *N. Engl. J. Med.*, **293**, 1339.

GASPARD, U., HENDRICK, J. C., REUTER, A. M. & FRANCHIMONT, P. (1973) Dosage radioimmunologique de l'hormone chorionique somatomammotrophe humaine (HCS) par les immunoadsorbants —anticorps: Son application à la clinique obstètricals et à la recherche des sécrètions hormonales ectopiques. *Ann. Biol. Clin. (Paris)*, **31**, 447.

GREENWOOD, F. C., HUNTER, W. M. & GLOVER, J. S. (1963) The preparation of $^{131}$I labelled human growth hormone of high specific radioactivity. *Biochem. J.*, **89**, 114.

HAAGENSEN, C. D. (1971) Adenofibroma of the breast. In *Diseases of the Breast*. London: Saunders. p. 212.

MCMANUS, M. J., DOMBROSKE, S. E., PIENKOWSKI, M. M. & 5 others (1978) Successful transplantation of human benign breast tumors into the athymic nude mouse and demonstration of enhanced DNA synthesis by human placental lactogen. *Cancer Res.*, **38**, 2243.

MIDGLEY, A. R., JR (1966) Radioimmunoassay: A method for human chorionic gonadotrophin and human luteinizing hormone. *Endocrinology*, **79**, 10.

PAYNE, R. A. & RYAN, R. J. (1972) Human placental lactogen in male subject. *J. Urol.*, **107**, 99.

ROSEN, S. W., WEINTRAUB, B. D., VAITUKAITIS, J. L., SUSSMAN, H. H., HERSMAN, J. M. & MUGGIA, F. M. (1975) Placental proteins and their subunits as tumor markers. *Ann. Intern. Med.*, **82**, 71.

SHETH, N. A., SURAIYA, J. N., RANADIVE, K. J. & SHETH, A. R. (1974) Ectopic production of human chorionic gonadotrophin in human breast tumours. *Br. J. Cancer*, **30**, 566.

SHETH, N. A., SURAIYA, J. N., SHETH, A. R., RANADIVE, K. J. & JUSSAWALLA, D. J. (1977) Ectopic production of human placental lactogen by human breast tumors. *Cancer*, **39**, 693.

WELSCH, C. W., DOMBROSKE, S. E. & McMANUS, M. J. (1978) Effects of insulin, human placental lactogen and human growth hormone on DNA synthesis in organ cultures of benign human breast tumours. *Br. J. Cancer*, **38**, 258.

WEINTRAUB, B. D. & ROSEN, S. W. (1971) Ectopic production of human chorionic somatomammotropin by non-trophoblastic cancer. *J. Clin. Endocrinol. Metab.*, **32**, 94.

YANAI, R. & NAGASAWA, H. (1973) Enhancement by human placental lactogen of mammary hyperplastic nodules in ovariectomized mice. *Cancer Res.*, **33**, 1642.